# Vaccinia virus and Cowpox virus are not susceptible to the interferon-induced antiviral protein MxA

**DOI:** 10.1371/journal.pone.0181459

**Published:** 2017-07-20

**Authors:** María M. Lorenzo, Juana M. Sanchez-Puig, Rafael Blasco

**Affiliations:** Departamento de Biotecnología, Instituto Nacional de Investigación y Tecnología Agraria y Alimentaria (I.N.I.A.), Madrid, Spain; The Scripps Research Institute, UNITED STATES

## Abstract

MxA protein is expressed in response to type I and type III Interferon and constitute an important antiviral factor with broad antiviral activity to diverse RNA viruses. In addition, some studies expand the range of MxA antiviral activity to include particular DNA viruses like Monkeypox virus (MPXV) and African Swine Fever virus (ASFV). However, a broad profile of activity of MxA to large DNA viruses has not been established to date. Here, we investigated if some well characterized DNA viruses belonging to the Poxviridae family are sensitive to human MxA. A cell line inducibly expressing MxA to inhibitory levels showed no anti-Vaccinia virus (VACV) virus activity, indicating either lack of susceptibility of the virus, or the existence of viral factors capable of counteracting MxA inhibition. To determine if VACV resistance to MxA was due to a virus-encoded anti-MxA activity, we performed coinfections of VACV and the MxA-sensitive Vesicular Stomatitis virus (VSV), and show that VACV does not protect VSV from MxA inhibition *in trans*. Those results were extended to several VACV strains and two CPXV strains, thus confirming that those Orthopoxviruses do not block MxA action. Overall, these results point to a lack of susceptibility of the Poxviridae to MxA antiviral activity.

## Introduction

Mx proteins are induced as part of the antiviral response triggered by type I and type III interferons. They belong to a family of large GTPases that includes dynamin, but despite this similarity, their detailed molecular mechanism of action is currently not known [1]. Some of the Mx proteins have been shown to exert antiviral activity against a wide range of RNA viruses. In the best studied example, mouse Mx1 inhibits influenza virus infection by blocking viral transcription and replication [2]. The antiviral range of activity of different Mx proteins seems to depend largely on the subcellular localization of the protein. Thus, nuclear forms (like mouse Mx1) protect against viruses that replicate in the cell nucleus, while cytoplasmic forms (like mouse Mx2) inhibit replication of VSV and some other viruses that replicate in the cytoplasm.

Human MxA and MxB proteins have been shown to have distinct antiviral activities. While MxB restricts HIV-1 and similar lentiviruses by inhibiting nuclear import [1, 3–6], human MxA has a broad activity against many diverse RNA viruses [1, 2, 7]. Also, MxA inhibits Hepatitis B virus replication by inhibiting the nuclear export of viral RNAs [8]. In addition to those activities, MxA has been reported to have inhibitory activity against two large DNA viruses like African Swine Fever virus (ASFV) [9] and Monkeypox virus (MPXV) [9, 10], although no precise inhibitory mechanism has been proposed.

To date, the range of action of MxA proteins is only partially established. Further, no common theme for the inhibition of positive strand RNA, negative strand RNA and DNA-containing viruses is evident. Here, we have studied the susceptibility of Vaccinia virus (VACV) and Cowpox viruses (CPXV) to MxA antiviral action, and concluded that these viruses are not affected by the antiviral action of MxA. Also, we provide data indicating that VACV and CPXV do not provide a *trans-acting* activity counteracting MxA action.

## Materials and methods

### Cells and viruses

BSC-1 cells (ATCC CCL-26) were grown in Eagle’s Minimal Essential Medium (EMEM) supplemented with 0.1mg/ml penicillin, 0.1mg/ml streptomycin, 2 mM L-glutamine (BioWhittaker) and 5% fetal bovine serum (FBS). Flp-In^™^ 293 T-REx cells (Invitrogen-Life Technologies), derived from 293 human embryonic kidney cells, were grown in Dulbecco’s minimal essential medium (DMEM) supplemented with 0.1mg/ml penicillin, 0.1mg/ml streptomycin, 2 mM L-glutamine (BioWhittaker) and 7% fetal bovine serum (FBS). Cells were maintained in medium containing 5 μg/ml Blasticidin (Invivogen) and 300 μg/ml Zeocin (Invivogen). MxA-293T cells were grown in Dulbecco’s minimal essential medium (DMEM) supplemented with 0.1mg/ml penicillin, 0.1 mg/ml streptomycin, 2 mM L-glutamine (BioWhittaker) and 7% fetal bovine serum (FBS) 5 μg/ml Blasticidin (Invivogen) and 100 μg/ml Hygromycin (Invivogen).

VSV-ΔG was kindly provided by Brian Lichty, McMaster University, Canada [11] [12]. VSV-GFP [13] was obtained from Sean P. J. Whelan, Harvard medical school USA. CPXV BR and EP4 were obtained from A. Alcamí (Centro de Biologia Molecular, Madrid).

### Isolation of inducible cell line

An inducible cell line expressing MxA was isolated following the original protocols for the Flp-In T-Rex system (Invitrogen). Human MxA gene was obtained by PCR from plasmid pCMV-SPORT6-MX1 (Open Biosystems, Mammalian Gene Collection (MGC) Clone ID 5535278, sequence accession BC032602) with oligonucleotides Mx1-Nhe EcoRI F (5´-GCTAGCAGATTCAAAGAAGG-3´) and Mx1 HindIII NotI R (5´-AAGCTTAGCGGCCGCTACCCG-3´). This PCR fragment was digested with NheI and NotI and inserted between the corresponding restriction sites in plasmid pcDNA5/FRT/TO to obtain plasmid pcDNA-FRT-Mx1.

MxA-293T cells, containing a single inducible copy of the MxA gene, were isolated from 293-Flip-In T-Rex cells. Cells grown to 25% confluence on six-well plates were transfected with plasmids pcDNA-FRT-Mx1 and pOG44 (ThermoFisher Scientific) at a ratio of 1:3. At 18 hours post infection, cultures were transferred to a T25 flask y and incubated in DMEM– 7% FBS containing 100 μg/ml hygromycin until cell clones were visible. In all cases, more than 20 clones were present before cells were expanded as a mixed population.

Flag-PKR-293T cell line, expressing a Flag-tagged version of human PKR [14] was obtained following the same protocol using the plasmid pcDNA5/FRT/PKR, generously provided by Ju-Tao Guo (Drexel University, USA).

### Isolation of VACV recombinants

Recombinant VACV were isolated following the standard protocol of plasmid transfection of infected cells to allow recombination. BSC-1 cells were infected with VACV at a MOI of 0.05 PFU/cell and transfected 1 h later with insertion plasmids containing VACV recombination sequences. DNA transfection was carried out using Fugene transfection reagent HD (Promega) following manufacturer instructions. Recombinant viruses were isolated from progeny virus by rounds of plaque purification on BSC-1 cells, during which plaques were screened for plaque formation and/or fluorescence. The isolation of a stable double cross-over virus was verified by gpt assay in viruses derived from vRB10 and vRB12, or by fluorescence and/or PCR in the case of viruses derived from other viruses (not shown).

Virus recombinant V-A3r was obtained by infecting cells with VACV strain WR and transfecting with plasmid pBS-SKII-RFP-A3L [15], courtesy of M. Way. The recombination places RFP coding sequence in-frame with the A3L gene. Stable virus recombinants were selected using fluorescence screening and plaque selection.

Virus WI-ΔF13L-g was obtained from VACV WI [16] by substituting the F13L gene by the GFP coding sequence. With this aim, a recombination plasmid (prsGFP) was obtained by deleting the F13L open reading frame from plasmid pRB-rsGFP [17]. The left recombination flank, was amplified from pRB21 by PCR with oligonucleotides LLF13L (5’-GCATATGCATGCTTTGTTAAAATAGATA-3´, SphI underlined) and LRF13L (5'-CATTTTGCTCGAGCAGGTACCGATGCAA-3´, XhoI underlined). This sequence was inserted between the SphI and XhoI restriction sites in pRB-rsGFP to remove the F13L gene.

VACV recombinants V-MxA and WI-MxA, which express the MxA gene in the WR and WI [16] backgrounds were obtained from vRB12 and WI-ΔF13L-g respectively, by inserting the human MxA gene downstream of the F13L gene.

Plasmid pRB-MxA was constructed by isolating a EcoRI—HindIII fragment from plasmid pcDNA-FRT-Mx1, containing the MxA gene and inserting this fragment between the corresponding EcoRI/HindIII of plasmid pRB21 [18].

Poxvirus recombinants expressing BFP were obtained by transfecting plasmid pRB-LF-TagBFP (containing a TagBFP cassette cloned downstream of the F13L gene in pRB21 plasmid, MML, manuscript in preparation) into cells infected with VACV vRB12 [18], MVAΔF13L [19, 20], vRB10 [21], CPXV Brighton red, or CPXV EP4 to isolate WR-BFP, MVA-BFP, IHDJ-BFP, BR-BFP and EP4-BFP, respectively. Virus isolation was accomplished by plaque identification aided by BFP fluorescence and plaque purification.

### Virus infection and titration

Virus infections were carried out in media containing 2% FBS. To induce expression, cells grown in six-well plates were incubated with medium containing 1 μg/ml tetracycline for 18h prior to infection. After infection, cells were maintained in EMEM containing 2% FBS. Viral titers in cell lysates obtained after disrupting the cells by three rounds of freeze/thawing were determined by the standard VACV plaque assay in BSC-1 cells [22]. Infectivity assay for VSV was performed by plaque assay on cells under EMEM-2% FBS containing 0.75% methylcellulose. VSV titers were always obtained from clarified cell culture medium, where VACV titers are significantly lower, and were distinguished morphologically from VACV plaques after staining with crystal violet solution (0.5% w/v crystal violet, 20% v/v ethanol).

To measure plaque size, BSC-1 cell monolayers in 6-well plates were infected with 20–50 PFU to generate well isolated virus plaques. After 48 hours, monolayers were fixed and stained with crystal violet solution. Plates were photographed, and the area of thirty plaques per virus was quantitated using ImageJ software.

### Western blotting

After infection or treatment, cell extracts were prepared in denaturant buffer (80 mM Tris-HCl, pH 6.8, 2% sodium dodecyl sulfate [SDS], 10% glycerol, 0.01% bromophenol blue solution and 0.71 M 2-mercaptoethanol). After SDS-polyacrylamide gel electrophoresis (PAGE), proteins were transferred to PVDF membranes and incubated overnight at 4°C with primary diluted antibody in PBS containing 0.05% Tween-20 and 1% nonfat dry milk. Primary antibodies were Rabbit polyclonal anti-Mx1antibody (Santa Cruz Biotechnology, ref SC-50509) diluted 1:500; Rat monoclonal antibody 15B6, specific for F13 (made available by G. Hiller) diluted 1:50; monoclonal anti-Actin (Sigma ref:A4700) diluted 1:500 and rabbit anti-GFP polyclonal antibody (Chemicon ref:AB3080) diluted 1:1000. After extensive washing with PBS-0.05% Tween-20, membranes were incubated with HRP-conjugated secondary antibodies diluted 1:3000. Secondary antibodies were polyclonal goat anti-rabbit IgG (Dako P0448), polyclonal goat anti-rat IgG antibody (Dako P0450) and goat anti-Mouse polyvalent immunoglobulins (Sigma ref: A0412). After removal of unbound antibody, membranes were incubated for 1 min with a 1:1 mix of solution A (2.5 mM luminol [Sigma], 0.4 mM ρ-coumaric acid [Sigma], 100 mM Tris-HCl, pH 8.5) and solution B (0.018% H_2_O_2_, 100 mM Tris-HCl, pH 8.5) to finally record the luminiscence using a Molecular Imager Chemi Doc-XRS (Bio-Rad). The quantification of the bands was performed using the program Image Lab 3.0.1 (Bio-Rad).

### Immunofluorescence microscopy

Cells grown on round coverslips in 24-well plates were washed twice with PBS and fixed by the addition of ice-cold 4% paraformaldehyde for 12 min. All subsequent incubations were carried out at room temperature. Cells were permeabilized by a 15 min incubation in PBS containing 0.1% Triton X-100. Cells were treated with PBS containing 0.1 M glycine for 5 min and incubated with primary antibodies diluted in PBS–20% FCS for 30 min followed by incubation with secondary antibodies diluted 1:400 in PBS-20% FCS. Antibodies used were rat monoclonal antibody 15B6 (anti-F13) diluted 1:50, anti-Mx1antibody (Santa Cruz Biotechnology, ref SC-50509) diluted 1:200, anti-rat IgG—Alexa Fluor 594, anti-mouse IgG—Alexa Fluor 488 (Invitrogen). Finally, cells were washed extensively with PBS, mounted with FluorSave reagent (Calbiochem), and observed by fluorescence microscopy.

DNA was stained with Hoechst by incubating cell on glass coverslips with 2 mg/ml bisbenzimide (Hoechst dye. Sigma) for 30 min. To stain with To-Pro-3 (ThermoFisher ref T3605), permeabilized cells were incubated for five minutes in a 1:500 dilution of the commercial 1mM stock in PBS.

### Flow cytometry

MxA-293T cells grown in six-well plates were pre-treated for 18h with 1 μg/ml tetracycline prior to infection to induce MxA expression. Subsequently, cells were infected with VSV-GFP or coinfected with the different Orthopoxviruses at an MOI of 3 pfu/cell in EMEM—2% FCS. After 24h, the cells were detached from the plastic by trypsin treatment, collected in EMEM—2% FCS and sedimented by low-speed centrifugation. Resuspended cells were washed with phosphate-buffered saline (PBS) and fixed for 15 min at room temperature with 200μl of ice-cold 4% paraformaldehyde in PBS. Finally, 300 μl of PBS containing 0.1%BSA was added. Flow cytometry was done in a FACSCanto II cytometer (BD Biosciences, San Diego, CA) and data were processed with BD FACS Diva software (BD).

## Results

### VACV growth is not inhibited by MxA

**Fig 1:**
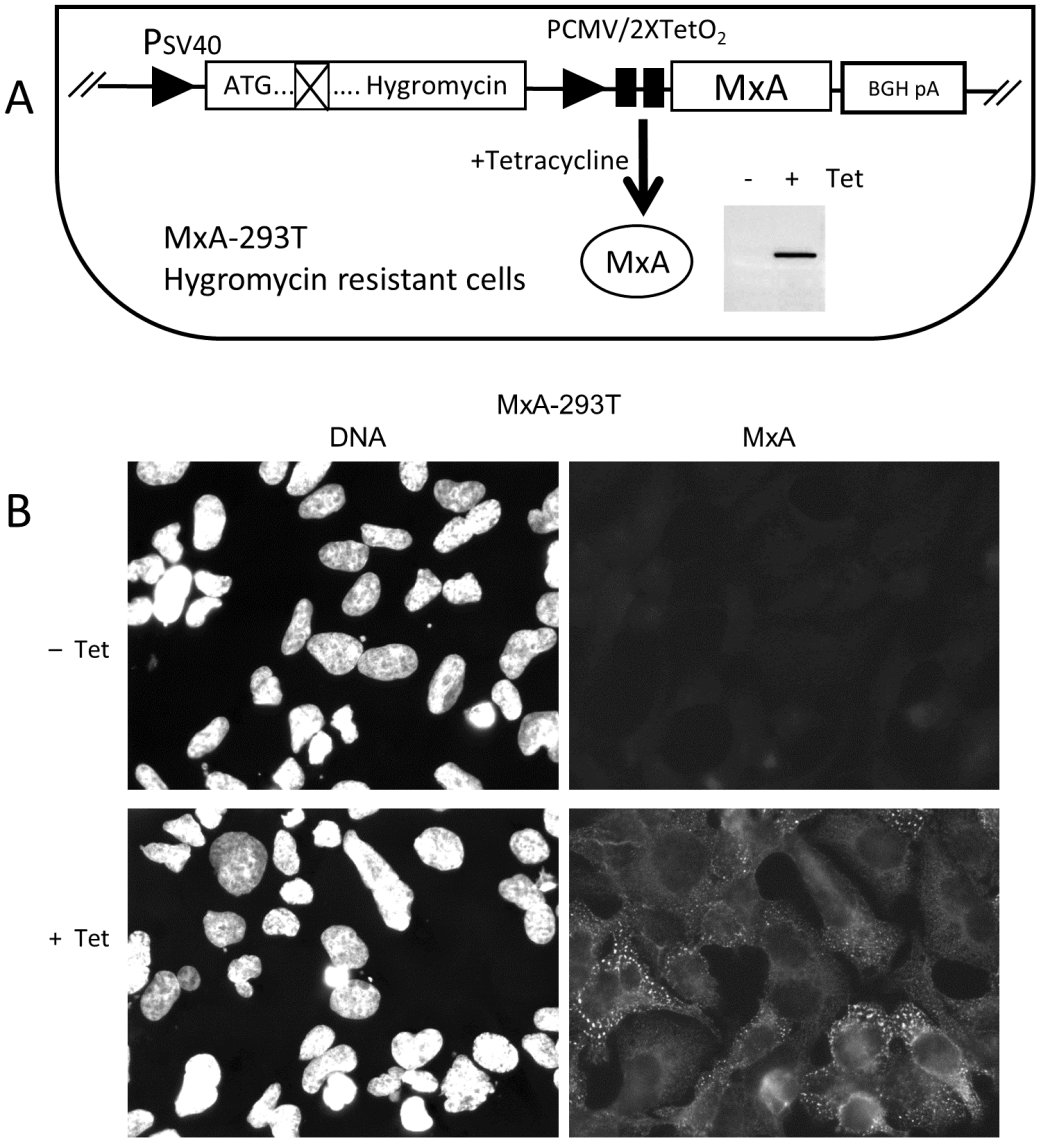
Isolation and characterization of the MxA-293T cell line. A) Scheme showing the insertion site for the MxA gene. Insertional recombination of the plasmid results in a functional Hygromycin resistance gene, and places MxA gene under the control of a CMV promoter / 2xTet operator cassette (PCMV/2xTetO_2_). Insert shows a western blot on cell extracts showing MxA accumulation after induction with medium containing 1 μg/ml tetracycline for 18h (+Tet). B) Subcellular distribution of MxA. DNA (Hoechst) staining and MxA staining are shown for uninduced (-Tet) and induced (+Tet) cell cultures.

In order to study the effect of MxA expression on VACV replication, we constructed a cell line capable of inducibly expressing human MxA. To avoid clone to clone variation, which can be significant [23, 24] we chose a system devised to generate uncloned isogenic cells. A cell population derived from human 293 cells was generated by insertion of the MxA gene, was named MxA-293T and was characterized in detail. Upon Tet induction, MxA protein was expressed by these cells and showed the characteristic intracellular distribution previously described for MxA (see [24, 25]) as revealed by staining with specific antibodies (Fig 1). Notably, basal MxA levels in uninduced cell cultures were undetectable by immunofluorescence or western blot analysis.

**Fig 2:**
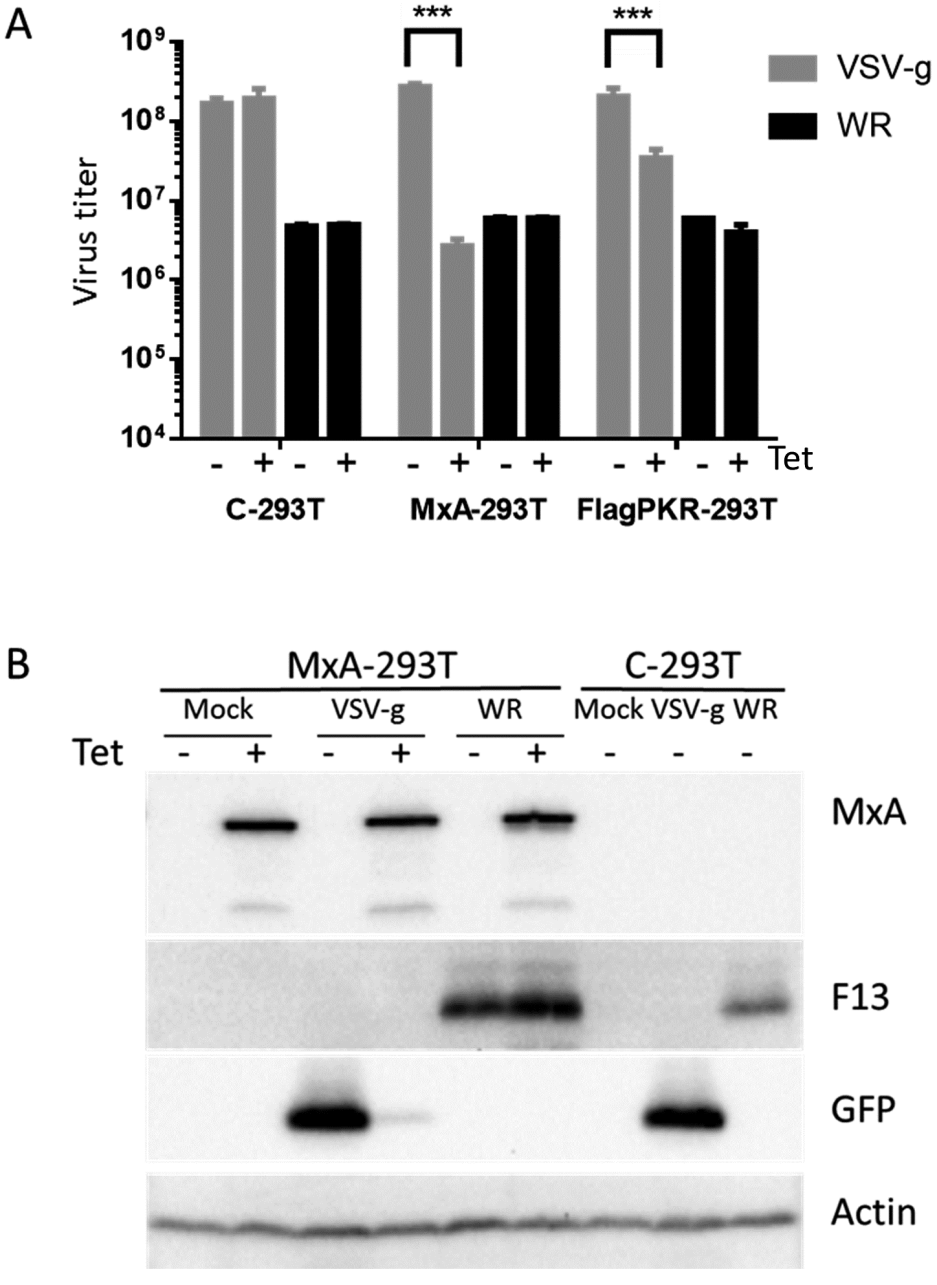
MxA expression in MxA-293T does not inhibit VACV replication. Cells were incubated for 18h with medium containing 1 μg/ml tetracycline and subsequently infected with vaccinia virus strain WR (WR) or VSV-GFP recombinant (VSV-g) at an MOI of 3 pfu/cell. A) Virus production at 24 h. Total VACV titers (cell+medium) or VSV titers (medium) were determined by plaque infectivity assay. C-293T are the parental FITR-293T cells. As a positive control, a similar cell line expressing a flag-tagged version of PKR (FlagPKR-293T) was included. Note: this tagged version of PKR was less active in VSV inhibition than the non-tagged version (M.L., unpublished). B) Extracts obtained from the cells indicated above were subjected to immunoblot with the antibodies indicated on the right of each panel. Note the drastic decrease in GFP expression in cells infected with VSV-g as a consequence of MxA expression, and the normal expression of a VACV late protein (F13).

To test the effect of MxA on VACV infection we carried out infections of MxA-293T cells, and determined progeny virus titers and protein expression (Fig 2). VACV replication levels and gene expression were not significantly affected by MxA expression, and were similar to those obtained with the control parental cell line. In contrast, the MxA-sensitive Vesicular Stomatitis Virus suffered a 2.5 log reduction in progeny virus titer and a 20 fold inhibition in gene expression, in agreement with previous reports [24, 26]. Those results indicate that MxA protein expressed by MxA-293T cells was functional, and that VACV growth was not sensitive to the antiviral activity of MxA.

**Fig 3:**
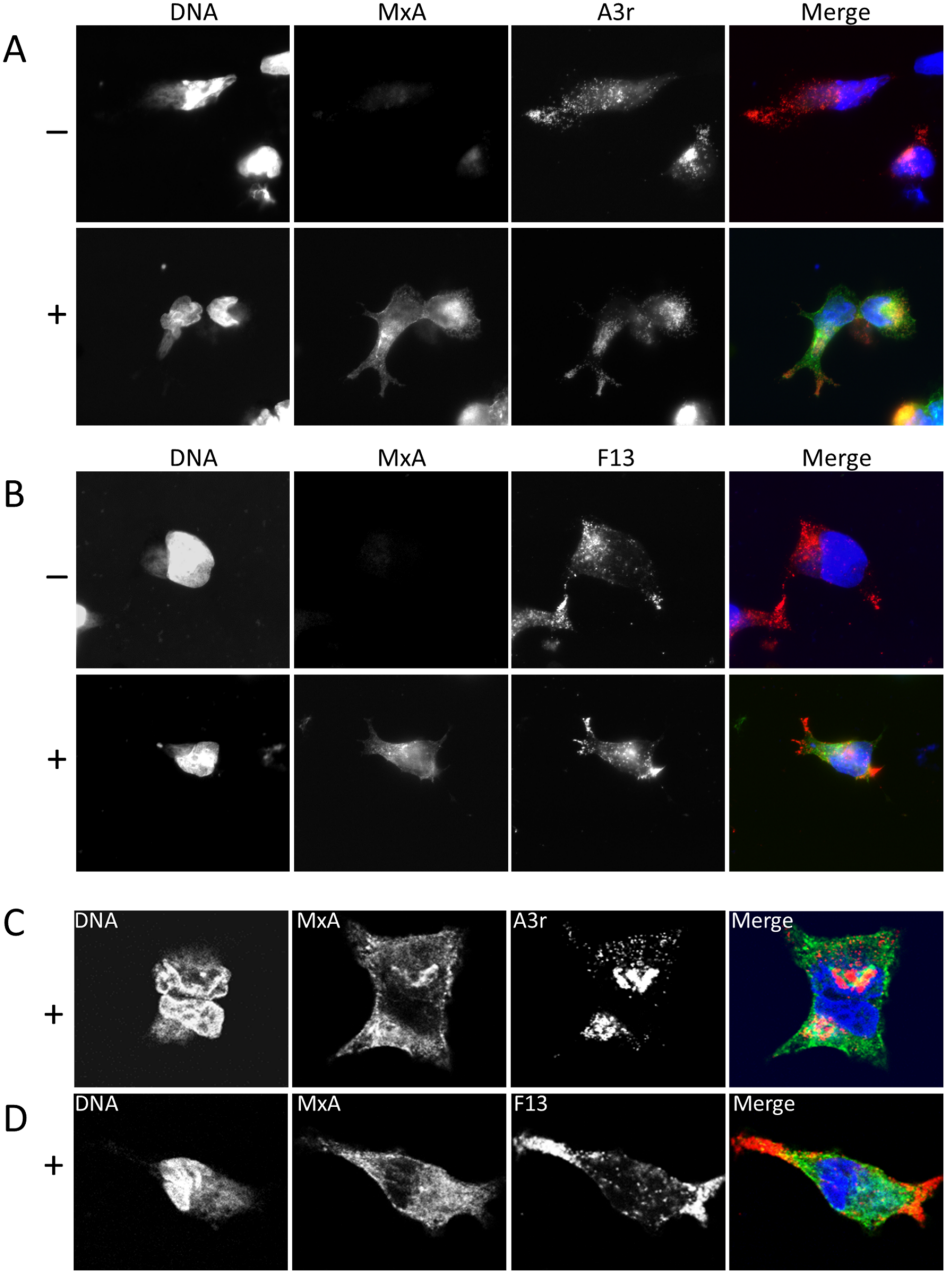
Subcellular distribution of MxA in infected cells. Cells were infected for 18 hours with VACV recombinant expressing RFP- tagged A3 protein to label viral factories and VACV Intracellular Mature Virions (A, C) or VACV strain WR (B,D). Cells were stained with To-Pro3 to visualize DNA, anti-MxA antibody or anti-F13 antibody, as indicated. Tet induction (“+” rows) was allowed for 18 hours before infection with medium containing 1 μg/ml tetracycline. A and B are widefield microscopy images, C and D are confocal images.

In agreement with the normal replication levels in induced MxA-293 cells, the intensity and distribution of viral proteins F13 and A3 were not significantly altered in infected cells in the presence of MxA (Fig 3). However partial redistribution of MxA as a consequence of infection was noted, since instead of the granular staining seen in non-infected cells, an increase in the perinuclear labeling was apparent (Fig 3). This staining partially overlapped with the area of viral factories, and was close but did not overlap with the TGN area, as revealed by A3 and F13 staining, respectively.

### VACV infection does not rescue VSV from MxA inhibition in coinfected cells

**Fig 4:**
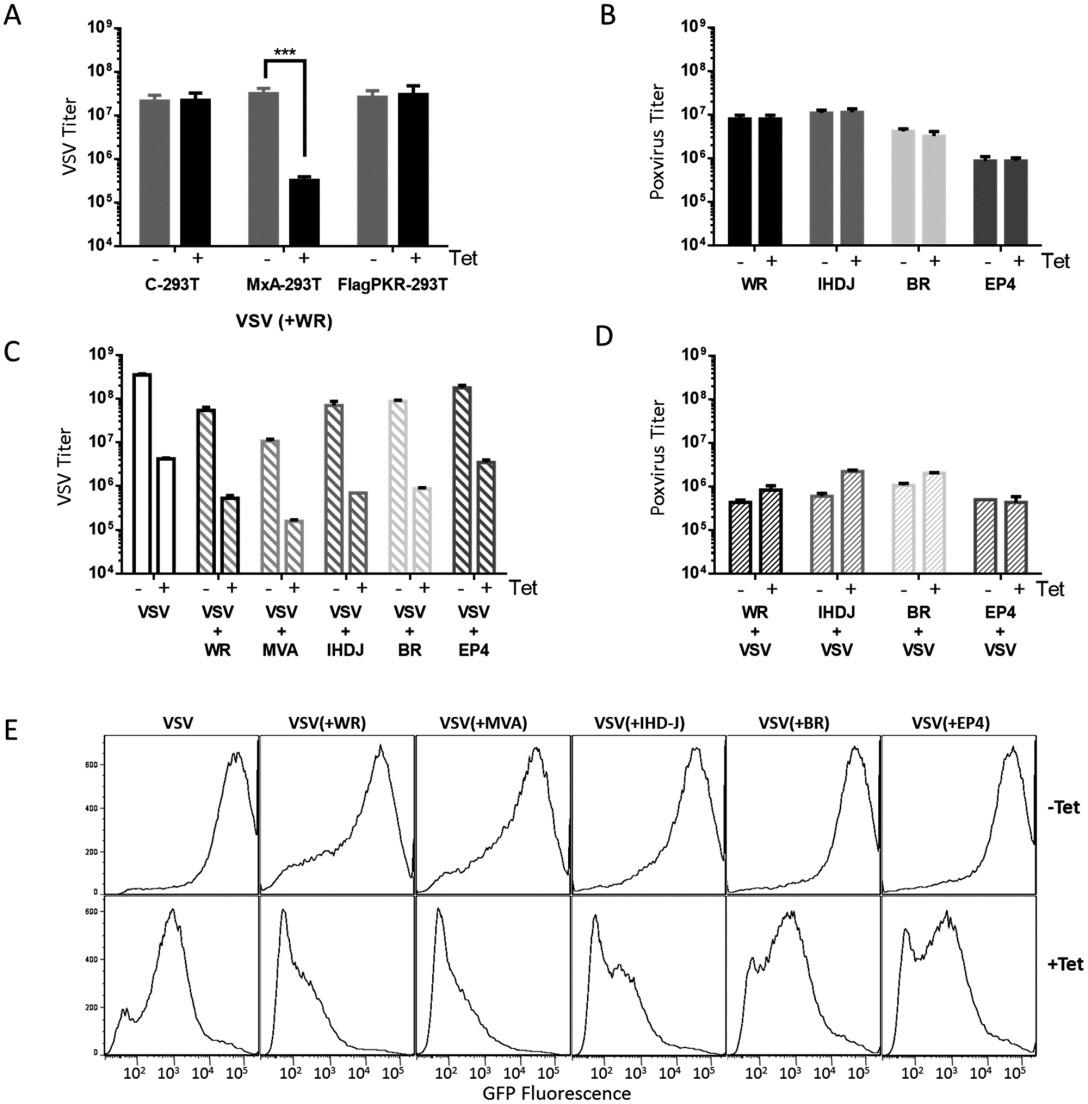
Lack of an anti-MxA trans-acting factor in Orthopoxviruses. A) Coinfection of VSV and VACV strain WR in parental control cells (C-293T), cells inducibly expressing MxA (MxA-293T) or cells inducibly expressing Flag-tagged PKR (FlagPKR-293T). Titers of VSV in the culture medium at 24 h are shown. B) Uninduced (-) or Tet induced (+) MxA-293T cells were infected with the Orthopoxviruses indicated, and incubated for 24 h. Orthopoxvirus production in cell lysates is shown. C, D) growth of VSV and Poxviruses in coinfected MxA-293T cells. For each infected culture, infection was allowed for 24 hours, and titers of VSV were obtained from the culture medium (C) and Poxvirus titers from cell lysates. Note the slight increase in replication of the Poxviruses when the competing VSV is inhibited by MxA. E) Flow cytometry analysis of VSV-g GFP fluorescence in cells coinfected with Orthopoxviruses. The Orthopoxviruses used were VACV strains WR, MVA and IHD-J, and CPXV strains BR and EP4.

The resistance of VACV replication to MxA could derive from an inherent resistance of the virus, or could alternatively be the result of an active blockage of MxA action by a yet unknown virus induced activity. To test this possibility, we designed experiments to detect a VACV *trans-acting* factor that might potentially counteract MxA antiviral activity. To this aim, we used a VACV-VSV coinfection assay that was previously used to detect VACV activities inhibiting PKR [27, 28]. In these experiments, after MxA induction for 18 hours, cells were subsequently coinfected with VACV and VSV and final virus titers produced at 24h for both viruses were determined by plaque assay (Fig 4A–4D). In coinfected cells, both VACV and VSV produced slightly less infectious titers than in single-infected controls, likely due to competition of the two viruses for cellular resources. As before, VACV replication was unaffected in MxA-expressing cells (Fig 4B). In contrast, VSV titers were reduced by MxA irrespective of the presence of coinfecting VACV in the same cells (Fig 4A). Therefore, VSV was sensitive to MxA action in VACV infected cells, indicating the absence of a trans-acting activity capable of blocking the anti-VSV effect of MxA. As a positive control, we performed parallel infections of a similar cell line expressing a flag-tagged version of PKR, in which VACV cross-protected VSV, presumably by the protein E3.

We also included in this analysis other members of the Orthopoxvirus genus, including VACV strains IHD-J and MVA and two CPXV strains, Brighton Red (BR) and EP4 (EP4). None of them were sensitive to MxA induction in MxA-293T cells (Fig 4B) or were capable of rescuing VSV replication from MxA inhibition (Fig 4C). In an analogous experiment, a VSV recombinant expressing GFP was used and, in addition to progeny virus, GFP fluorescence was analyzed by flow cytometry as a measure of viral gene expression (Fig 4E). As above, co-infection with an Orthopoxvirus was not able to protect VSV from MxA inhibitory action, symptomatic of the lack of an anti-MxA function in VACV and related Orthopoxviruses.

### VACV recombinants expressing MxA are viable and induce a functional anti-VSV activity

The above results indicate that pre-existing MxA does not preclude VACV infection in the MxA-293T cell line. However, it is conceivable that an effect of MxA on VACV infection during late times in the replication cycle might be prevented by VACV-induced changes on cellular gene expression, protein modifications and/or protein turnover. To rule out this possibility, we constructed VACV recombinants expressing MxA by inserting the MxA cDNA downstream of the F13L viral gene, in the VACV WR background and in WI virus that produces increased amounts of extracellular virus [16]. After infection/transfection, three independent virus clones were isolated and expanded. All three clones formed normal plaques and were able to grow to normal titers. One of such clones for each virus was further characterized.

**Fig 5:**
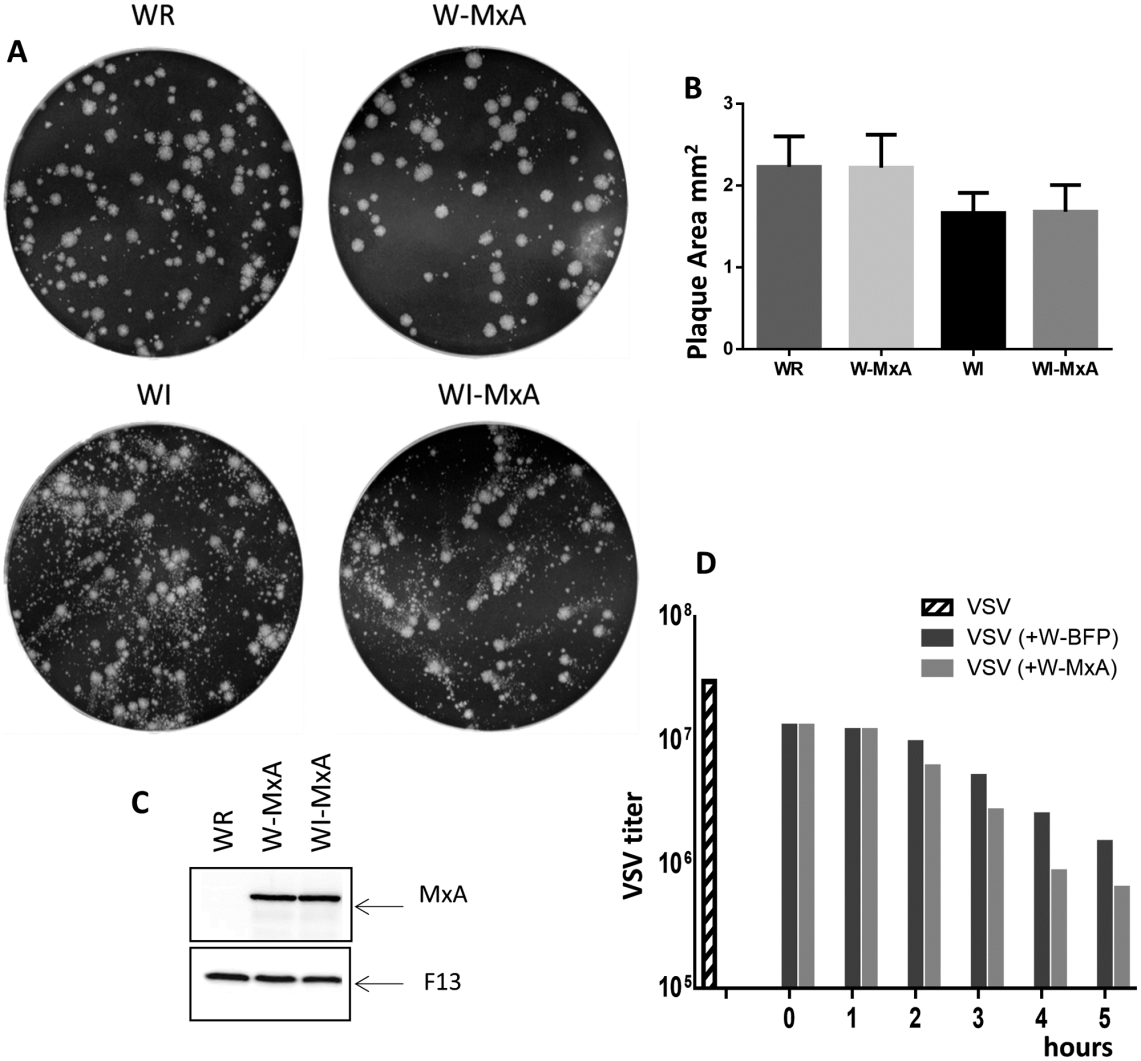
Characterization of MxA-expressing VACV recombinants. Virus recombinants W-MxA and WI-MxA contain the MxA gene under the control of a synthetic early/late promoter in the genetic background of virus strain WR or WI [16] respectively. A) Standard 2-day VACV plaque assay under liquid medium. B) Mean area of virus plaques made by different parental and recombinant viruses (n = 30). C) Immunoblot on cell extracts of cells infected by VACV (WR) or the VACV recombinants W-MxA or WI-MxA, revealed with antibodies to MxA or to the VACV late protein F13. D) Coinfection of VSV with VACV recombinants expressing MxA. To achieve some MxA expression before VSV replication, VSV infection was delayed with respect to VACV infection for the times indicated in the x axis. VSV titers from cultures coinfected with control VACV (W-BFP, dark grey bars) or MxA-expressing virus (W-MxA, light grey bars) are shown. Hatched bar corresponds to the VSV titer obtained in the absence of coinfecting VACV.

Replication levels of VV-MxA recombinants were similar to that of the control parental viruses, and the plaque size appeared unaffected (Fig 5). To confirm that MxA produced by VACV recombinants was functional, we tested the ability of MxA expressed from VV-MxA to inhibit VSV. Synchronized infection of VSV and VV-MxA did not result in VSV inhibition, probably reflecting the fast replication cycle of VSV, and the relatively slower kinetics of MxA expression by VACV. Thus, we sought to allow some MxA expression before the onset of VSV replication cycle by starting VSV infection at different times after VACV infection (Fig 5D). Under those conditions, the maximum effect of MxA was obtained when VSV was added 4 hours after the onset of VACV infection, where VSV titers were reduced about twofold with respect to the coinfection with control VACV (Fig 5D) indicating that MxA expressed by VV-MxA was able to inhibit VSV replication.

**Fig 6:**
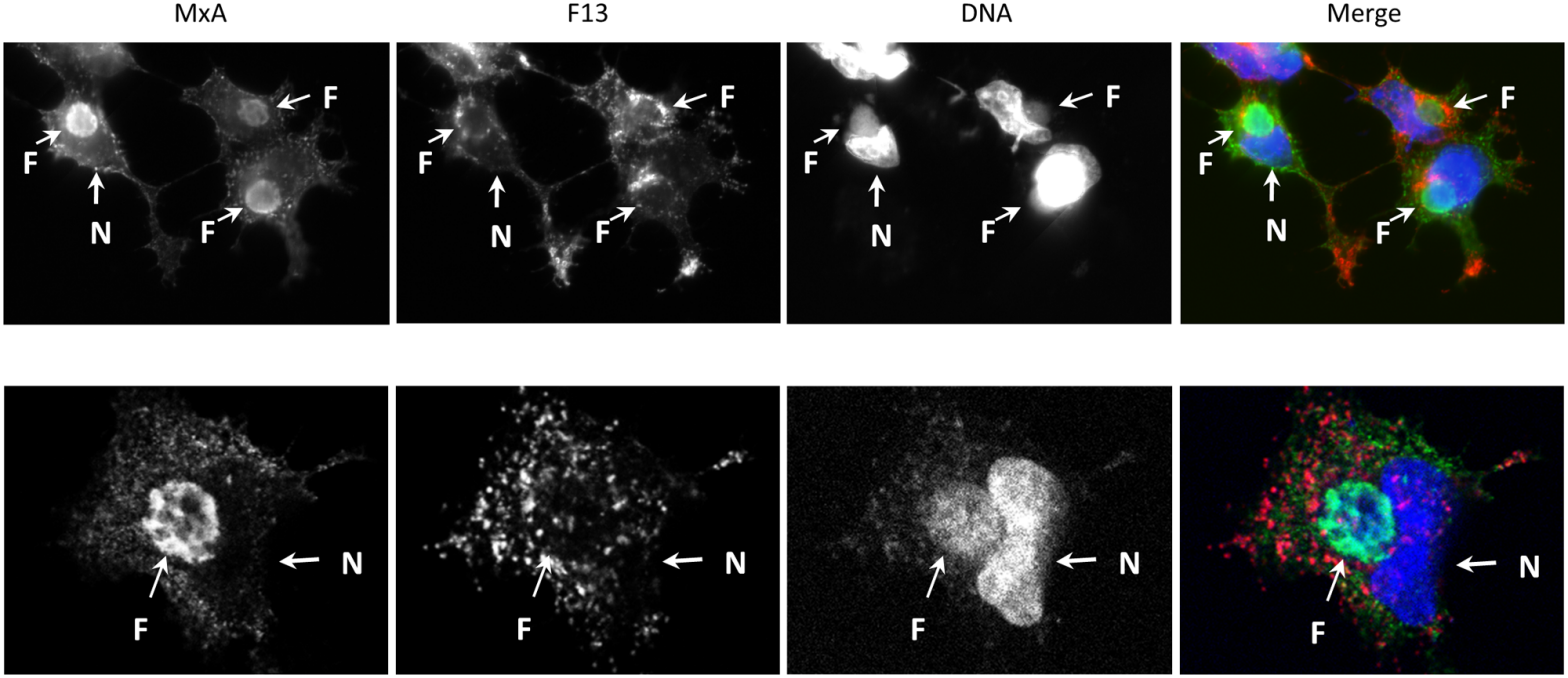
Intracellular localization of MxA in cells infected with recombinant virus VV-MxA. Fluorescence microscopy images obtained by widefield (A) or confocal (B) microscopy in C-293T cells infected with VV-MxA for 18 hours. The images shown correspond to MxA, F13 and DNA staining. In the Merge panel, MxA, F13 and DNA monochrome images were pseudocolored as green, red and blue and combined together. The position of viral factories (F) is indicated by arrows. The brighter DNA staining corresponds [5] to cell nuclei (N).

Since specific redistribution of MxA in cells infected with ASFV and MPXV have been reported, we considered of interest to determine the location of MxA expressed from VV-MxA recombinant. Notably, in addition to a cytoplasmic dispersed staining, there was a significant MxA signal overlapping with viral factories (Fig 6). However, no enrichment of MxA was detected in the Golgi complex area, as revealed by F13 staining. Overall, those results confirmed the absence of an inhibitory effect of MxA towards VACV replication, even in conditions of MxA overexpression and enrichment of the protein in viral factories.

## Discussion

Although the antiviral activity of MxA protein to many RNA viruses has been studied in detail, the cases of MxA activity reported towards DNA viruses are scarce and details about the inhibitory mechanism against large DNA viruses are basically unknown. In the best studied examples, the inhibition of RNA viruses by Mx proteins has been shown to rely on oligomerization of Mx molecules induced by viral ribonucleoprotein complexes. While this mode of action may explain the activity of Mx proteins to many RNA viruses, a common viral feature accounting for MxA activity against the distant DNA viruses has not been found. In this work, we have aimed at extending the characterization of the antiviral activity range of MxA, and provide evidence of the lack of susceptibility of a model large DNA virus (VACV) and related Orthopoxviruses to MxA.

Here, we have shown that MxA, expressed from the cell or even overexpressed from the viral genome, does not affect VACV replication. The resistance of VACV to MxA is not unexpected, since VACV is able to grow in cells pretreated with IFN-I, that express MxA together with many other Interferon-Stimulated Genes.

We considered the possibility that resistance of VACV, and other Orthopoxviruses, to MxA action might be attained by a virally-induced blockage of MxA antiviral activity. This would be in line with multiple studies that indicate that VACV resistance to IFN is the result of the expression of a pleyade of virally encoded proteins that counteract ISG action (reviewed in [29]). Thus, we tested the hypothesis that VACV encodes an MxA counteracting factor(s) by a classical VACV—VSV coinfection assay [30, 31] where VACV provides protection to VSV in Interferon-treated cells and has been useful before to map VACV-encoded *trans-acting* factors [28].

Some previous observations support the lack of activity of Mx proteins towards Poxviruses. For instance, VACV mutants with a deleted in the E3L gene, and that are sensitive to IFN, are recovered by knocking down the PKR gene but not MxA [32], suggesting that the well characterized poxviral protein E3L is not related to MxA activity. Also, studies of virus susceptibility in the mouse model indicate that rodent Mx proteins are not important in the control of Poxviral infections, while they are crucial in Influenza virus infection. Most laboratory strains of mice carry an inactive Mx1 gene, and this deficiency is related to a high susceptibility to Myxoviruses like Influenza Virus [33–35]. However, some wild-type strains of mice carrying Mx1 genes show enhanced susceptibility to MPXV infection, whereas Mx1-negative laboratory strains of mice such as BALB/C are highly resistant [36, 37]. Although rodent Mx proteins differ in activity and intracellular localization from human MxA, these observations therefore point to factors other than Mx proteins in determining susceptibility of mice to Orthopoxviruses, and are in general agreement with our results.

Our data diverge from previous reports showing susceptibility of large DNA viruses to MxA, including ASFV [9] and the Orthopoxvirus MPXV [10]. One case that needs consideration is that of MPXV, which is closely related to both VACV and CPXV. Similar to VACV, MPXV has been reported to be resistant to IFN [38] even though it lacks a fully functional E3 gene that counteracts several ISGs. Our data indicate that the reported susceptibility of MPXV to MxA [10] is not paralleled in VACV or CPXV. There are several possibilities to explain the different behavior of such closely related viruses. First, genetic differences between MPXV and other Orthopoxviruses could account for these disparities, similar to other cases where related viruses have distinct susceptibility to MxA. Such a situation has been reported for two Rhabdoviruses where Rabies virus, in contrast to VSV, is not affected by MxA expression [39]. However, alternative explanations exist. The discrepancies related to MPXV susceptibility to MxA inhibition may be derived from the cell lines and/or cell clones used in these studies. In the report by Johston et al, 2012 [10], sensitivity was studied with cloned Vero cells [40] that differ with the inducible, syngenic cell system used here. In this respect, when isolating clones from established cell lines, we have repeatedly observed clone-to-clone variation in VACV production (not shown). Notably, ASFV susceptibility was studied with the same, or related, Vero cell clones [9]. Extended studies on additional cell lines and viruses may be required to further clarify the observed range of MxA inhibitory activities in different cell-virus systems.

We have shown an alteration of the intracellular distribution of MxA as a result of infection, with partial overlap with viral factories, but this redistribution was not accompanied by inhibition of the virus replication. MxA is relocalized around DNA-containing viral factories in ASFV-infected cells, which represent virus assembly sites. In contrast, in MPX infected cells, MxA was shown to colocalize partially with the virus wrapping area, but not with viral factories [10]. Our results are reminiscent of the distribution reported for ASFV infection since partial colocalization with the viral factories area was detected. MxA protein associates with membranes and is seen as small dots or granules in the cytoplasm of stably transfected mouse 3T3 or Vero cells [41, 42] although has been shown to display different intracellular distribution depending on the level of expression, and to colocalize with markers for the smooth ER, [25]. One interesting possibility is that MxA may be enriched in VACV assembly areas as a consequence of the recruitment of ER membranes to viral factories, during the virion assembly process (reviewed in [43]).
